# Desiccation tolerance in the chlorophyte green alga *Ulva compressa*: does cell wall architecture contribute to ecological success?

**DOI:** 10.1007/s00425-015-2292-6

**Published:** 2015-04-21

**Authors:** Andreas Holzinger, Klaus Herburger, Franziska Kaplan, Louise A. Lewis

**Affiliations:** Institute of Botany, Functional Plant Biology, University of Innsbruck, Sternwartestrasse 15, 6020 Innsbruck, Austria; Department of Ecology and Evolutionary Biology, University of Connecticut, Storrs, CT 06269-3043 USA

**Keywords:** Cellulose, Imaging PAM, Intertidal zone, Macroalgae, Pectin, Photosynthesis, *rbc*L phylogeny

## Abstract

**Desiccation leads to structural changes of the inner pectic cell wall layers in*Ulva compressa*. This contributes to protection against mechanical damage due to desiccation–rehydration cycles.**

*Ulva compressa*, characterized by *rbc*L phylogeny, is a common species in the Mediterranean Sea. *Ulva* as an intertidal species tolerates repeated desiccation–rehydration cycles in nature; the physiological and structural basis were investigated under experimental conditions here. Desiccation to 73 % relative water content (RWC) led to a significant decrease of the maximum quantum yield of photosystem II (*F*_v_/*F*_m_) to about half of the initial value. A reduction to 48 or 27 % RWC caused a more drastic effect and thalli were only able to recover fully from desiccation to 73 % RWC. Relative electron transport rates were stimulated at 73 % RWC, but decreased significantly at 48 and 27 % RWC, respectively. Imaging-PAM analysis demonstrated a homogenous desiccation process within individual thallus discs. The different cell wall layers of *U. compressa* were characterized by standard staining procedures, i.e. calcofluor white and aniline blue for structural components (cellulose, callose), ruthenium red for pectins and toluidine blue for acidic polysaccharides. Already a reduction to 73 % RWC caused severe changes of the cell walls. The inner pectin-rich layers followed the shrinkage process of the cytoplasm, while the outer denser fibrillar layers maintained their shape. In this way, the thalli were not plasmolyzed during water loss, and upon recovery not negatively influenced by any mechanical damage. Transmission electron microscopy corroborated the arrangement of the different layers clearly distinguishable by their texture and electron density. We suggest the flexibility of the pectin-rich cell wall layers as a major contribution to desiccation tolerance in *Ulva*.

**Electronic supplementary material:**

The online version of this article (doi:10.1007/s00425-015-2292-6) contains supplementary material, which is available to authorized users.

## Introduction

Desiccation tolerance (DT) is a relatively rare yet phylogenetically widespread phenomenon in green algae; however, the strategies to achieve tolerance are only partly understood. Among green algae, DT occurs in the chlorophyte as well as in streptophyte lineages, the latter giving rise to land plants (Leliaert et al. 2012). Among the chlorophyte lineage, many DT green algae are photobionts of lichens (Kranner et al. 2005; Gasulla et al. 2013) or free-living terrestrial algae (Gray et al. 2007). Many physiological traits have been investigated in Chlorophyta, providing a good background to understand physiological performance under water-limited conditions (Holzinger and Karsten 2013; Karsten and Holzinger 2014).

Intertidal species of algae have to tolerate desiccation along with drastic variation in salinity (Smith and Berry 1986; Kirst 1990) requiring a high (i.e. very negative) osmotic potential and water-holding capacity. Ionic relations were studied in *Ulva*, where an accumulation mechanism for potassium was found, while sodium was actively excluded (West and Pitman 1967).

*Ulva* Linnaeus (Ulvophyceae) is an emerging model organism for green macroalgae and much effort has been made to investigate physiological reactions involved in tolerance of the colonization of the intertidal zone (Gao et al. 2011). Different *Ulva* sp. have been investigated concerning their ability to tolerate various stress scenarios including UV irradiation (Franklin et al. 1992; Bischof et al. 2002a, b; Rautenberger and Bischof 2006) and osmotic stress (Dickson et al. 1982; Xia et al. 2004; Gao et al. 2014). Recently, transcriptomes of *Ulva* became available, providing a better understanding of the genetic basis underlying stress tolerance (Zhang et al. 2012). Many inquiries have focused their investigations on photosynthetic parameters in this alga, driven by incredible algal blooms (green tides) e.g. in the Yellow Sea or the north-western coast of France (Briand 1991; Gao et al. 2010; Smetacek and Zingone 2013). The global success of *Ulva* has motivated detailed investigations of the cellular mechanisms associated with such incredible physiological performance.

Some peculiarities of the photosynthetic apparatus have been detected, including selective targeting of PSII upon desiccation, whereas PS I has the astonishing capacity to tolerate water loss under exposure to even 4 M sorbitol (Gao et al. 2014). This sorbitol concentration translates into an osmotic potential of ~−23.25 mPa (Kosugi et al. 2014). The capacity of the photosynthetic apparatus to tolerate water loss accompanied by salinity stress during low tide followed by rapid recovery during high tide is astonishing. But this physiological performance, which is also investigated in the present study in *Ulva compressa*, cannot be the only explanation for the success of *Ulva* sp. on a global scale.

The aim of the present study, to investigate the role of cell wall composition and structure on the ability of *Ulva* to cope with water stress, was motivated by the observation that in nature these intertidal algae remain visibly intact. Cell walls of Ulvophyceae contain cellulose, β-mannans, xyloglucan, β-xylans, sulfated polysaccharides (ulvan), homogalacturonan, rhamnoxylogalacto-glucuronan and arabinogalactan proteins (Abdel-Fattah and Edrees 1972; Percival 1979; Lahaye and Ray 1996; Lahaye and Robic 2007; Ray 2006; Estevez et al. 2009; Ciancia et al. 2012; Domozych et al. 2012) and even the occurrence of lignin has been considered (Yaicha et al. 2011).

There is much literature available on ulvan, a sulphated polysaccharide in Ulvophyceae, which can comprise up to 29 % of algal dry weight (Lahaye and Robic 2007). Like other sulphated polysaccharides, ulvan is considered an adaptation to marine habitats (Aquino et al. 2011). Ulvan was mainly investigated in terms of its usefulness for food, pharmaceutical, agricultural and chemical applications (Lahaye and Robic 2007; Wijesekara et al. 2011; Ciancia et al. 2012), and its linkage to negative impact on coastal ecosystems (Wang et al. 2011). However, to our knowledge, no studies deal with the structural behaviour of the cell wall in *Ulva* under desiccation stress. Little is known about the ability of *Ulva* cell walls to plasticize, which is considered to be important for surviving desiccation in some green algae and resurrection plants (Holzinger et al. 2011; Moore et al. 2013). In resurrection plants, the pectin matrix is thought to play a key role in maintaining cell wall plasticity during desiccation stress by preventing irreversible polymer adhesion. In the present study, we provide the first investigation of the role of pectin-rich cell wall layers in plasticising the cell walls of *Ulva*.

We visualize matrix components (e.g. pectins) of the cell wall by different staining procedures and follow their change during experimental desiccation. Furthermore, we characterized the fibrillar components of *Ulva* cell walls by general cell wall stains. Our hypothesis is that the pectic compounds contribute to a regulated shrinkage process in the event of desiccation, and this process was therefore analysed by transmission electron microscope (TEM) as well as light microscopic techniques. Moreover, a detailed physiological description of desiccation effects in *Ulva* was performed. To estimate the effect of desiccation on a cellular level, a microscopic version of an Imaging PAM was used to answer the questions whether the thalli desiccated homogenously or an inhibition of photosynthesis first occurs in individual cells. Furthermore, we performed a profound physiological description of DT reactions in *U. compressa*, one of the most common *Ulva* species in Europe (Blomster et al. 1998). In addition to the morphological species determination, the alga was analysed by *rbc*L phylogeny.

## Materials and methods

### Strain origin and culture conditions

*Ulva compressa* was collected on April 8, 2012 at a beach near Sousse, Tunisia (35°51′34.9″N 10°37′02.9″E). Thalli were washed in sea water to remove sediment and transferred to the laboratory, where unialgal cultures were established. Algae were cultivated in 250–1000 mL Erlenmeyer flaks containing artificial seawater (ASW; Schweiger et al. 1977). They grew in a dark/light regime of 14:10 h in a modified refrigerator (Liebherr AG, Bulle, Swiss) equipped with Osram Daylight Lumilux and Fluora lamps (L36W/77, L36W/827; Osram, Munich, Germany) at 16 °C and ~40 μmol photons m^−2^ s^−1^ in the light period. The lamps were mounted on the outside of this culture chamber to avoid excessive heating.

### DNA extraction and phylogenetic analysis

DNA was isolated using the PowerPlant DNA Isolation Kit (Mo Bio Laboratories, Inc., Carlsbad, CA, USA) according to the manufacturer’s protocol. Primers and PCR conditions used for PCR amplification and cycle sequencing of *rbc*L are listed in McManus and Lewis (2011). Individual sequence reads were inspected for quality and then used to prepare a consensus, which was then aligned manually with published *rbc*L sequences of related *Ulva* taxa. The appropriate substitution model was chosen using jModeltest v0.1 (Posada 2008) under the Akaike Information Criterion (AIC). Phylogenetic analysis under the maximum likelihood (ML) criterion was performed in PAUP* (Swofford 2002), using a GTR + I + ${\varvec{\Gamma}}$ model, with parameter values estimated during the ML heuristic search. Bootstrap analysis was performed under the same model, except that parameter values were set based on the ML tree. Bayesian analyses were run in MrBayes v.3.2.1 (Huelsenbeck and Ronquist 2001; Ronquist and Huelsenbeck 2003) for 5 × 10^6^ generations with one cold chain and three heated chains, using two parallel runs, under the GTR + I + ${\varvec{\Gamma}}$ model. Trees were sampled every 1000 generations. The first 10 % of samples were discarded as burnin. Parameter stability and run convergence were inspected using Tracer v1.4.1 (Rambaut and Drummond 2003).

### Desiccation experiment and determination of the relative water content

Six discs were cut out with a punch (diameter = 6 mm) from mid-sized thalli, excess ASW was removed from the surface of the discs. The discs were placed in a desiccation chamber according to Karsten et al. (2014); the relative humidity (RH) of ~62 % was adjusted by a 7 M lithium chloride solution (Sigma-Aldrich, Steinheim, Germany). Relative humidity was recorded using a PCEMSR145STH mini data logger (PCE Instruments, Meschede, Germany). The chamber was placed under a halogen lamp (~40 μmol photons m^−2^ s^−1^ PAR) at room temperature (22 ± 0.5 °C).

Six *U. compressa* thallus discs were weighed after removal of surface water with a paper towel and subsequently placed in a desiccation chamber under the same conditions as described above. Discs were weighed again after 30, 60 and 90 min to determine the weight of the desiccated discs. Then, they were dried in the oven at 100 °C for 13 h and weighed again, allowing calculating the relative water content (RWC):

$${\text{RWC (}}\% )= \frac{(W_t - W_d)}{(W_0 - W_d)}\;\times\text{100}\;\%,$$

where *W*_*0*_ is the weight of the fully hydrated discs, *W*_*t*_ is the weight of discs at time *t* after desiccation and *W*_*d*_ is the weight of the oven-dried discs. 30 min of desiccation corresponded to a RWC of 73 ± 4 %. After 60 and 90 min, RWC was reduced to 48 ± 5 % or 27 ± 4 %, respectively (cf Fig. S1).

### Light microscopy

Macroscopic images were taken using a Nikon Coolpix 8400 camera (Nikon Corp., Tokyo, Japan) connected to a Nikon SMZ800 stereomicroscope. Furthermore, samples were investigated by a Zeiss Axiovert 200 M microscope, equipped with a 63 × 1.4 NA objective lens. Cellulose within the cell walls was stained with 1 % calcofluor white (CFW; Sigma-Aldrich; Krishnamurthy 1999) and visualized by exposure to UV light (340–389 nm). Samples were stained with 0.1 % aniline blue (AB; Sigma-Aldrich) to determine if the walls contained callose.

To visualize pectic substances in the cell walls, a drop of 0.005 % ruthenium red (RR; Sigma-Aldrich) was added to a fresh or desiccated thallus piece on a slide, respectively, and subsequently investigated by light microscope (Stancheva et al. 2014). Additionally, semithin sections (~0.6 µm) from chemically fixed material (fixation as described below) were prepared with a Leica ultramicrotome (Leica Microsystems GmbH, Wetzlar, Germany) and stained with 0.3 % toluidine blue (TB; Holzinger et al. 2011).

### Determination of the pigment content

The pigment content was determined according to Lichtenthaler and Buschmann (2001). Briefly, discs (see section "Desiccation experiment and determination of the relative water content") from liquid culture (control) and 30, 60 and 90 min desiccated were extracted in 2 mL of acetone for 4 days at 4 °C in darkness and spectrophotometrically quantified.

### Transmission electron microscopy

For transmission electron microscopy and TB staining, *U.compressa* thallus discs were desiccated as described above. Control and desiccated discs were cut into pieces (2 × 2 mm) and prepared according to Holzinger et al. (2009). Briefly, thallus pieces were fixed for 1.5 h in 50 mM cacodylate buffer (pH = 6.8) containing 2.5 % glutaraldehyde followed by postfixation in 1 % osmium tetroxide for ~18 h at 4.6 °C. The pieces of thalli were rinsed, dehydrated in increasing ethanol concentrations and propylene oxide and embedded in modified Spurr’s resin. Preparation of ultrathin sections was performed with a Reichert Ultramicrotome, followed by counterstaining with 2 % uranyl acetate and Reynold’s lead citrate. Sections were examined with a Zeiss Libra 120 TEM (80 kV) connected to a ProScan 2 k SSCCD camera, controlled with OSIS iTEM software. Images were further processed with Adobe Photoshop (CS5) software (Adobe Systems, San Jose, CA, USA).

### Maximum photochemical quantum yield of PS II (*F*_v_/*F*_m_)

The maximum photochemical quantum yield of photosystem (PS) II (*F*_v_/*F*_m_ = *F*_m_ – *F*_o_/*F*_m_) during standardized desiccation treatment and rehydration was measured with a pulse-amplitude modulated fluorimeter (PAM 2500, Heinz Walz GmbH, Effeltrich, Germany) equipped with a red LED (630 nm). For measuring *F*_v_/*F*_m_, the discs were placed in a KS-2500 suspension cuvette (Heinz Walz GmbH) to allow dark adaptation (10 min) and ensure a uniform distance between each sample and the PAM light probe. *F*_v_/*F*_m_ was measured in control samples and after 30, 60 or 90 min of desiccation, respectively, followed by recovery in ASW for 2 h. After this time, the *F*_v_/*F*_m_ value of the recovered samples was determined.

### Imaging PAM

The microscopy version of an Imaging PAM (M-series, Heinz Walz GmbH) was used to visualize *F*_v_/*F*_m_ of PSII (false colour image) and near infrared remission (NIR, 780 nm) for the first time on a cellular level in *U. compressa*. Therefore, control and thalli discs desiccated as described above (30, 60 and 90 min) were dark adapted for 10 min on a slide, overlaid with two drops immersion oil and subsequently investigated under a modified Axio ScopeA.1 epifluorescence microscope equipped with a Zeiss Fluar 40 × 1.3 (∞/0.17) objective lens and a CCD Camera IMAG-K6 controlled with ImagingWinGigE (V2.45i) software. Measuring light for *F*_v_/*F*_m_ determination was provided by a LED (620 nm). Images were further processed with Adobe Photoshop (CS5) software.

### Measurements of relative electron transport rates

Relative electron transport rates (rETRs; Kromkamp and Forster 2003) as a function of 17 increasing light steps (3–2015 μmol photons m^−2^ s^−1^ PAR, each 30 s) were recorded using a PAM 2500. This was always performed independently in six replicates for control and after 30, 60 or 90 min desiccation treatment, respectively, to avoid distortion of rETR values in response to desiccation treatment due to high measuring light. Furthermore, discs independently desiccated for 30, 60 or 90 min were placed in ASW 2 h to estimate rETR of recovered samples. All measurements were performed on *U.compressa* thalli discs which were placed in a KS-2500 suspension cuvette without prior dark adaptation. Desiccation was performed in a chamber under the same conditions as described above. The rETR curves were fitted by the model of Walsby (1997) to derive three photosynthesis parameters: *α* positive slope at limiting photon fluence rates (electrons photon^−1^), *I*_k_ initial value of light-saturated photosynthesis (μmol photons m^−2^ s^−1^) and *rETR*_*max*_ maximum relative electron transport rate (μmol electrons m^−2^ s^−1^).

### Statistical evaluation of the data

*F*_v_/*F*_m_ values (*n* = 6) were determined in a time series (control, 30, 60 or 90 min desiccation and after rehydration) and rETR values (*n* = 6) were always measured in independent samples (control, 30, 60 or 90 min desiccation and in recovered samples). Chlorophyll *a*, *b* and carotenoid content (*n* = 4) were determined independently in control and after 30, 60 or 90 min desiccation. To analyse the influence of time on *F*_v_/*F*_m_ values, a repeated-measure analysis of variance (ANOVA) was performed, followed by a Tukey’s post hoc test (*P* < 0.001) to find significant differences among control, desiccated and recovered samples. Comparison of photosynthetic parameters derived from rETR measurements and chlorophyll *a*, *b* or carotenoid content was performed by one-way ANOVA, followed by Tukey’s post hoc test (*P* < 0.001) to find homogeneous subgroups of significantly different means. All statistical analyses were carried out in Origin 8.5 (OriginLab Corporation, Northampton, MA, USA).

## Results

### Molecular phylogeny

**Fig. 1:**
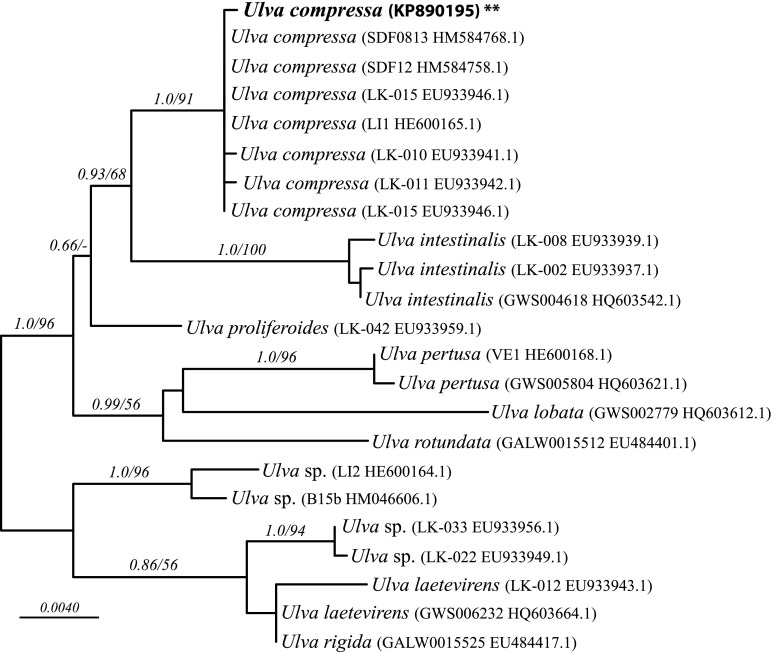
Phylogenetic tree of *U. compressa* used in this study plus published sequences of *U. compressa* and related species, based on a maximum likelihood analysis of *rbc*L data. Taxon labels include corresponding strain numbers and GenBank accessions numbers. Node support shown is based on ML bootstrap analysis followed by Bayesian posterior probabilities. *Bar* corresponds to expected number of substitutions per site

The *rbc*L sequence obtained from the *Ulva* sample was 1197 nucleotides long. The alignment of the new sequence with 22 published *Ulva* sequences was a total length of 1355 nucleotides, with no excluded, 1250 constant, and 70 parsimony-informative sites. The ML analysis yielded 24 optimal trees (ln L = − 2718.913) that differed only in the relative placement among sequences in a large clade corresponding to *U. compressa* (Fig. 1). The Bayesian analysis yielded a majority rule consensus tree that was congruent with the ML tree, so only the ML tree was illustrated. The new sequence was contained in a strongly supported *U. compressa* clade (1.0 posterior probability, 91 ML bootstrap), which was sister to *Ulva intestinalis*. Several other well-supported clades, representing different *Ulva* species, were also highly supported, in agreement with the analysis of Wolf et al. (2012).

### Macroscopic and microscopic aspects

**Fig. 2:**
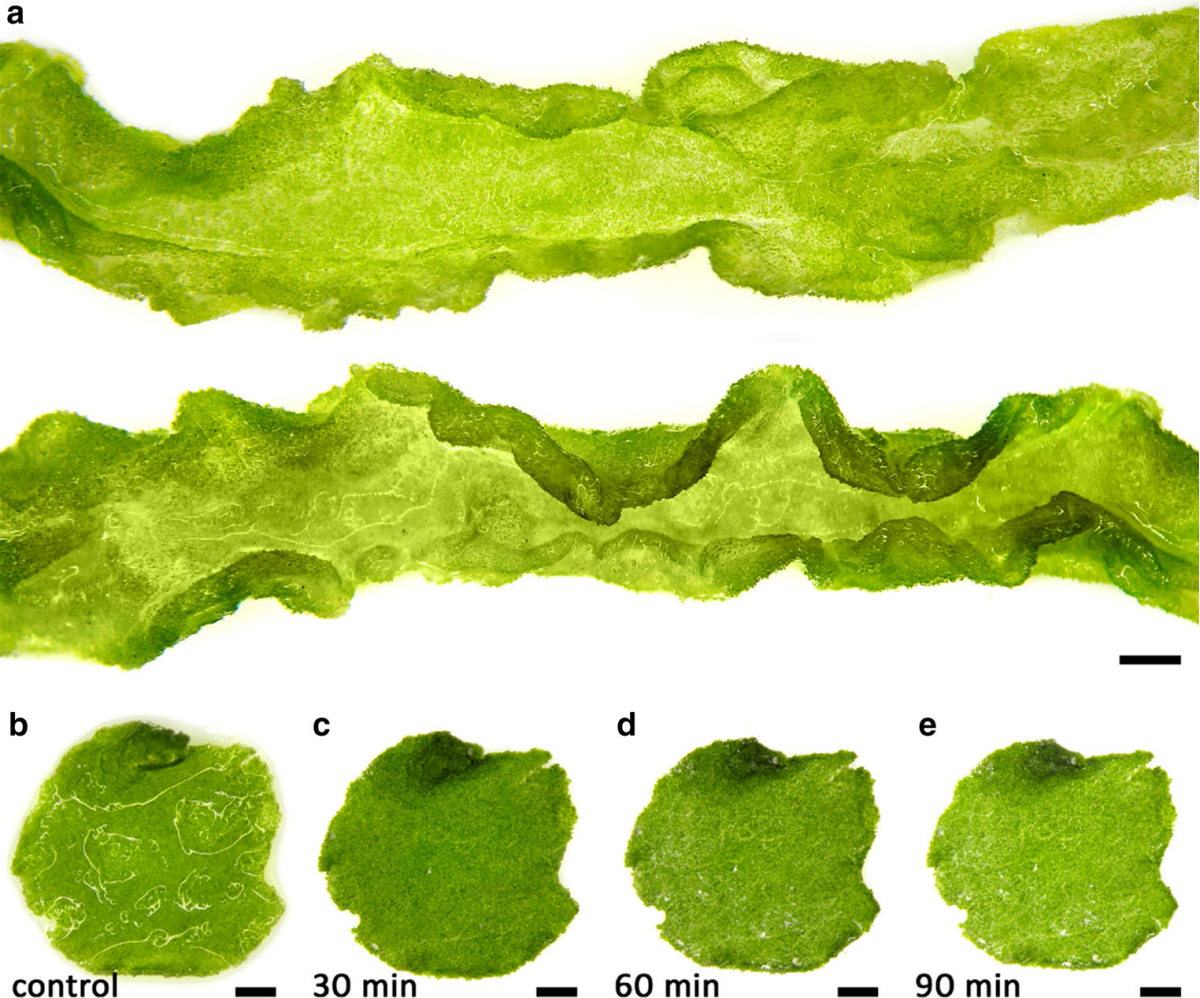
Macroscopic appearances of *U. compressa*. **a** Two thallus segments with typical conduplicated margins. **b**–**e** Exemplified thallus discs used for microscopic and physiological investigations in control conditions and after 30 min (73 % RWC), 60 min (48 % RWC) and 90 min (27 % RWC) of desiccation. While the control disc is covered with a thin water layer, after 30 min no surface water is visible. *Scale bars* 1 mm

**Fig. 3:**
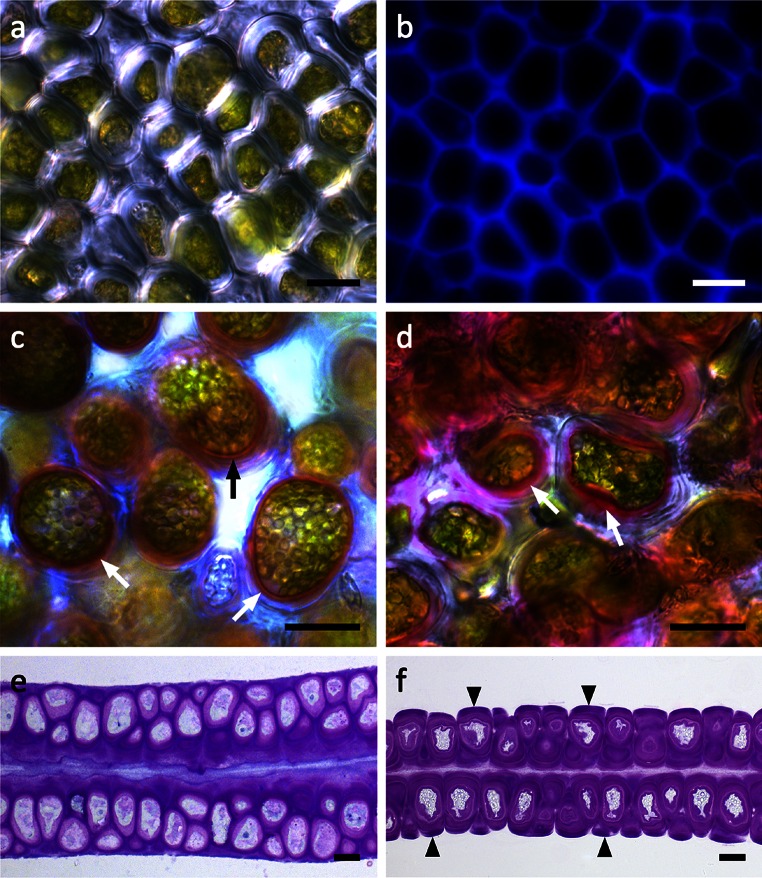
Light micrographs of *U. compressa*. **a** Surface view of a central area of the thallus. No intercellular spaces are visible between the polygonal cells. **b** Corresponding CFW staining of cellulose. **c** RR staining of fresh samples to visualize pectins, which are abundant in the inner cell wall layer attached to the protoplast (*arrows*). **d** RR staining in a sample desiccated for 30 min. The inner pectic-rich layer increases in diameter (*arrows*). **e** Chemically fixed, TB-stained semithin cross section of a fresh sample showing an irregular arrangement of cells in both thallus layers. TB-stained cross section of a sample desiccated for 30 min. Fragments of the cell wall matrix are attached to periclinal cell walls (*arrowheads*). The protoplasts show undulations followed by the cell walls, while no retraction of the cytoplasm from the cell walls occurs. The thickness of the thallus segment decreases. *Scale bars* 10 µm

*Ulva compressa* thalli were unbranched, tubular and showed typical conduplicated margins (Fig. 2a). Their diameter was between 4 and 10 mm, while mid-sized thalli were chosen to punch out discs for microscopic and physiological investigations (Fig. 2b–e). Control discs were covered with a thin water layer after removing most water with a paper towel (Fig. 2b). In contrast, after 30 min (73 % RWC) of desiccation at a relative humidity (RH) of ~62 %, no surface water was visible (Fig. 2b–e). Formation of salt crystals was scarce and the discs stayed visibly green (Fig. 2b–d). In top view, cells were irregularly arranged and had a diameter of 6–12 µm (Fig. 3a, b). Usually they were polygonal with rounded corners (Fig. 3a). However, cells were spherical when they became separated from the cell complex, as seen in some areas of the thallus (Fig. 3c, Fig. S2). The chloroplasts were hood-shaped or appeared to fill the cells (Fig. 3c). The thick multilayered cell wall contained cellulose (Fig. 3b) and pectic substances, which were particularly abundant in the innermost cell wall layer as shown by staining with RR and TB (Fig. 3c–f). This pectic layer increased in diameter after desiccation (Fig. 3d, f). Furthermore, cellular water loss led to shrinkage and undulations of the cell walls (Fig. 3f). In cross-sectional view, desiccated thalli appeared flatter compared to hydrated control groups (Fig. 3e, f).

### Pigment content

The pigment content decreased slightly in desiccated samples. Chlorophyll *a* was reduced significantly after 90 min desiccation from 38.88 ± 1.52 to 30.95 ± 1.81 mg m^−2^ (*n* = 4, *P* < 0.001, Fig. S3). Chlorophyll *b* was decreased from 25.87 ± 1.43 mg m^−2^ to 20.59 ± 1.14 mg m^−2^ in all desiccated samples, whereas the values for carotenoids did not change significantly upon desiccation (Fig. S3).

### Transmission electron microscopy

**Fig. 4:**
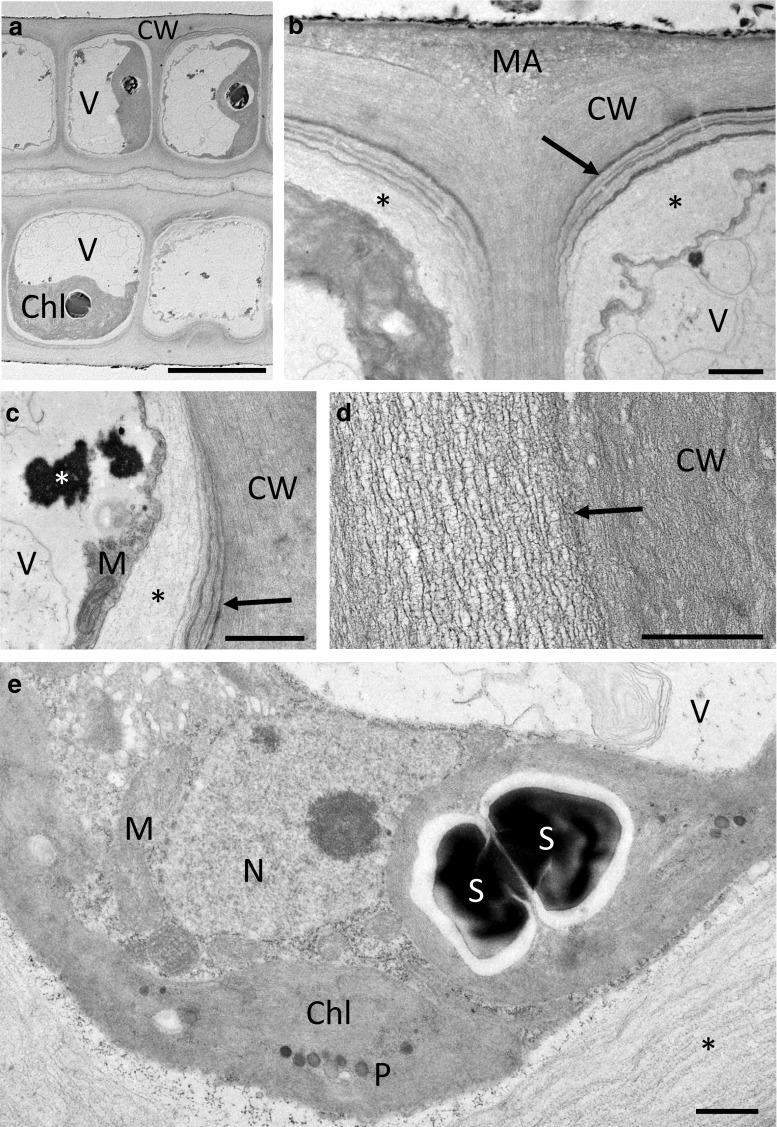
Transmission electron micrographs of the control of *U. compressa*. **a** Cross section through a thallus, where the cells contain a large vacuole, a nucleus and one large parietal chloroplast. **b** Detail of the layered cell wall between two individual cells. The individual cells are surrounded by dense fibrillar layers (CW) and enclosed by a pectic matrix (MA). The innermost cell wall layer consists of electron translucent pectic components (*asterisks*). Different layers in periclinal cell walls likely containing glucuronan are clearly visible (*arrow*). **c** Detail of the periclinal cell wall layers (*arrow*), flanking an electron translucent pectic layer (*black asterisk*). Cytoplasm closely attached to the cell wall contains mitochondria and electron-dense material (*white asterisk*). **d** Detail of the fibrillar structure of the cell wall composed out of layers of different density (*arrow*). **e** The chloroplast contains starch grains and plastoglobuli. The nucleus, mitochondria and endoplasmic reticulum are clearly visible. *Scale bars* 10 µm (**a**), 1 µm (**b**–**e**)

**Fig. 5:**
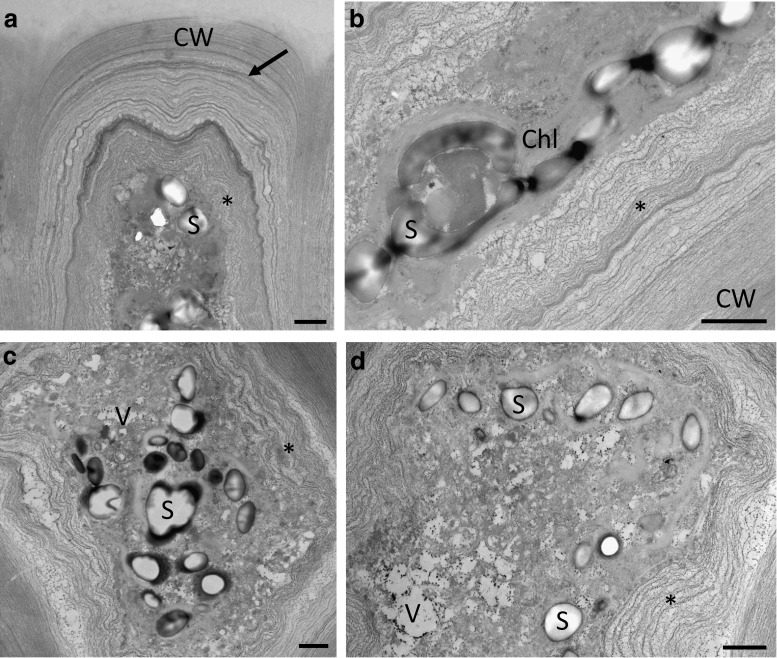
Transmission electron micrographs of desiccated (73 % RWC) and rehydrated (2 h in ASW) samples of *U. compressa*. **a** Desiccation leads to undulations of the inner cell wall layers and the cytoplasm appears dense due to shrinkage of the protoplast followed by the innermost pectic cell wall layers (*asterisk*). The outermost fibrillar cell wall layers surrounding a periclinal pectic layer (*arrow*) do not change in shape. **b** Detail of the cell wall after desiccation. The innermost pectic cell wall layers (*asterisk*) are attached to the shrunken protoplast. **c**, **d** Numerous stark grains and vacuoles are visible upon rehydration. Undulation of the inner pectic cell wall layers (*asteris*k) is still visible. *Scale bars* 1 µm

At the TEM level, the cellular architecture of cultivated cells under control conditions is illustrated in Fig. 4. The cells comprised a large central vacuole; the cytoplasm appeared pushed towards the periphery (Fig. 4a). The thallus consisted of two connected layers; the cells were connected by multilayered cell walls that are poreless (Fig. 4b–d). The outside of these walls was covered by electron-dense granules (Fig. 4b). The outer cell wall layers were very dense (Fig. 4b), followed by heterogeneous layers of varying electron density (Fig. 4b, d). Further towards the cytoplasm were electron translucent wall layers (Fig. 4b, c). These layers had different texture, varied in density, but were always clearly separated from the dense fibrillar structure of the outer cell wall layers (Fig. 4c). The cytoplasm contained the organelles in an expected arrangement: chloroplasts with starch grains, the nucleus, ER and mitochondria (Fig. 4e). After 30 min (73 % RWC) of desiccation at 62 % RH, substantial changes in the cell wall architecture as well as the cytoplasm were observed (Fig. 5a, b). While the cytoplasm appeared denser (Fig. 5a, b), the loose inner cell wall layers followed the shrinkage process of the cytoplasm, resulting in cells that were not plasmolysed (Fig. 5a, b). Upon recovery for 2 h in ASW, the cytoplasm contained numerous vacuoles and the undulated cell wall layers were still visible (Fig. 5c, d).

### Desiccation effects on *F*_v_/*F*_m_ and relative water content

**Fig. 6:**
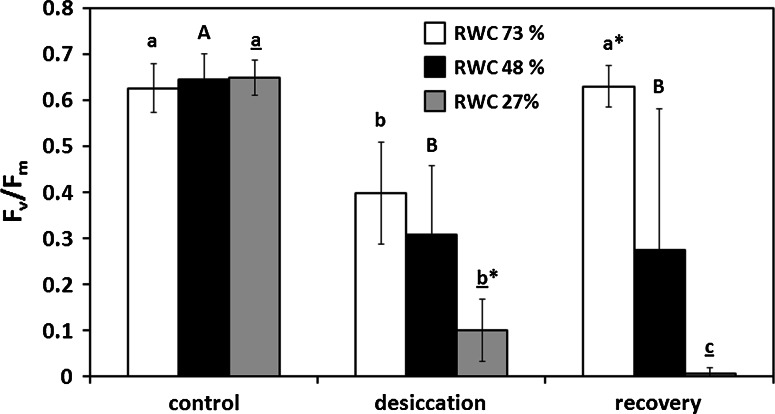
The maximum photochemical quantum yield (*F*_v_/*F*_m_) of PSII in response to controlled desiccation for 30 min (73 % RWC, *filled diamond*), 60 min (48 % RWC, *filled circle*) or 90 min (27 % RWC, *plus*), respectively (*n* = 6, mean value ± SD). For measuring recovery of *F*_v_/*F*_m_ after desiccation, samples were placed in ASW for 2 h. *F*_v_/*F*_m_ was estimated under ~40 μmol photons m^−2^ s^−1^ PAR. Significances between the treatments are indicated by *small letters* (30 min), *capital letters* (60 min) and *underlined letters* (90 min). *F*_v_/*F*_m_ values of control groups, different desiccation treatments or recoveries were compared and significant differences are marked with an *asterisk* (*n* = 6). Significances were determined by one-way ANOVA (*P* < 0.001) followed by Tukey’s post hoc test

The maximum photochemical quantum yield of photosystem II (*F*_v_/*F*_m_) was affected by experimental desiccation and showed significant differences depending on duration of desiccation (Fig. 6). Desiccation for 30, corresponding to a RWC of 73 %, and 60 min (48 % RWC) led to the same significant decrease of *F*_v_/*F*_m_ to 0.40 ± 0.06 and 0.31 ± 0.15, respectively (Fig. 6). However, full recovery after rehydration in ASW for 2 h was only achieved in discs with 73 % RWC (0.63 ± 0.04; Fig. 6), whereas in discs with 48 % RWC rehydration resulted in a significantly lower *F*_v_/*F*_m_ value compared to the control group (0.27 ± 0.30; Fig. 6). Desiccation of 90 min (27 % RWC) led to the strongest decrease of *F*_v_/*F*_m_ (0.10 ± 0.07), which did not recover upon rehydration (0.01 ± 0.01; Fig. 6).

### Imaging PAM

**Fig. 7:**
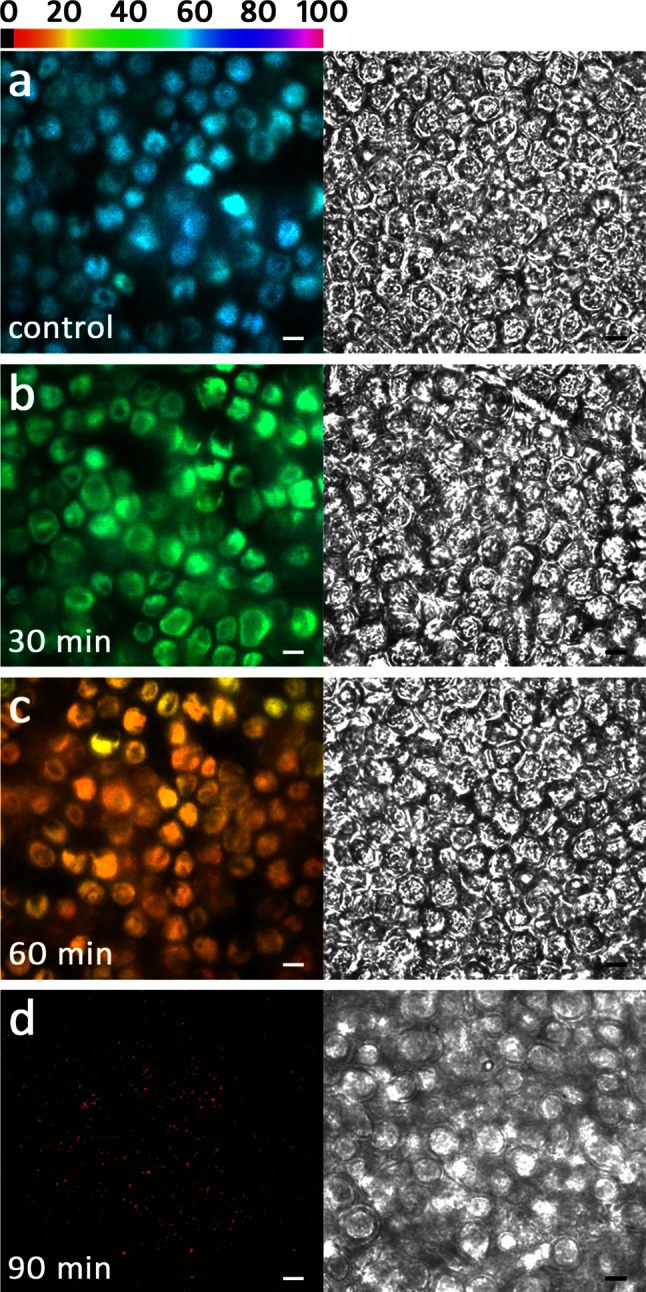
Visualized *F*_v_/*F*_m_ (*false colour image*, *left column*) and near infrared remission (NIR, *right column*) images. **a** Control group. **b** After desiccation for 30 min (73 % RWC), **c** 60 min (48 % RWC) and **d** 90 min (27 % RWC). *False colour code bar on top* represents the relative *F*_v_/*F*_m_ value as a percentage. *Scale bars* 10 µm

*F*_v_/*F*_m_ images recorded with an Imaging PAM always differed significantly between control discs and those with reduced RWCs (73, 48 and 27 %; Fig. 6a–d). The *F*_v_/*F*_m_ value of PSII in most chloroplasts of control samples was ~0.58 (Fig. 7a). In discs with 73 % RWC and 48 % RWC, it was reduced to ~0.46 (Fig. 7b) or ~0.13 (Fig. 7c), respectively, in agreement with the *F*_v_/*F*_m_ values measured with a PAM 2500 (Fig. 6). In discs with RWC 27 %, only diffuse signals were detectable, indicating *F*_v_/*F*_m_ values close to 0 (Fig. 7d). The corresponding NIR images always show the cellular organization of the investigated thallus area (Fig. 7a–d).

### Relative electron transport rates

**Fig. 8:**
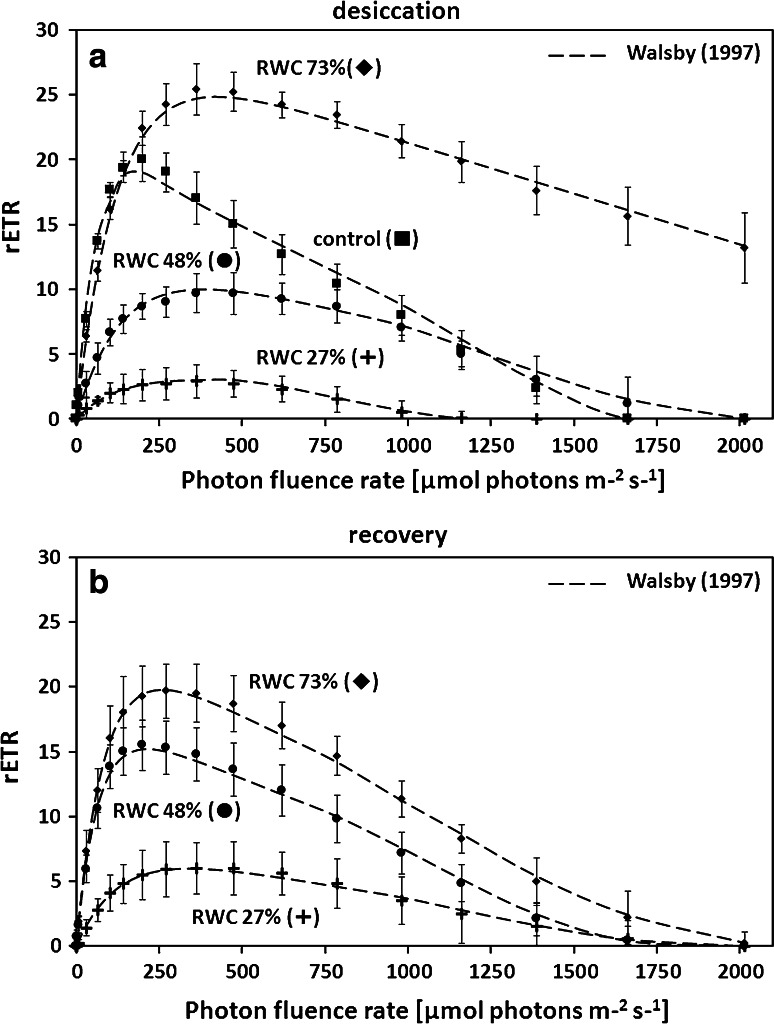
Relative electron transport rates (rETRs, μmol electrons m^−2^ s^−1^) as a function of increasing light intensities up to 2016 μmol photons m^−2^ s^−1^ in *Ulva* sp. **a** rETR curves in the control group and discs desiccated for 30 min (RWC 73 %), 60 min (48 %) or 90 min (RWC 27 %) at ~62 % RH (*n* = 6, mean value ± SD). **b** rETR curves in desiccated samples (30, 60 or 90 min) after 2 h recovery in ASW. rETR curves were determined using the fitting model of Walsby (1997) as photoinhibition occurred. Characteristic photosynthetic parameters (*α*, *I*_k_ and rETR_max_) derived from rETR curves are shown in Table 1

rETR as a function of increasing light intensities up to 2016 µmol photons m^−2^ s^−1^ showed strong differences between the different desiccation treatments and rehydration in ASW for 2 h (Fig. 8a, b; Table 1). The highest rETR_max_ values were measured in discs with 73 % RWC (Fig. 8a; Table 1). Significantly (*P* < 0.001) lower rETR_max_ values occurred in the control discs and discs with RWC 48 % and 27 % (Fig. 8a; Table 1). In contrast, increasing exposure to desiccation stress led to a continuous decrease of the *α* value (Table 1). This was accompanied by a significantly (*P* < 0.001) increasing *I*_k_ value in discs with RWC 73, 48 and 27 % (Table 1). Rehydrated discs with 73 % RWC showed the same rETR_max_ value as the control group, thus a significantly lower rETR_max_ compared to the value measured immediately prior rehydration (Fig. 8b; Table 1). In contrast, discs with 48 % RWC exhibited a significantly higher rETR_max_ value after rehydration, however, not reaching the values of control discs (Fig. 8b; Table 1). Rehydration of discs with 27 % RWC did not result in a recovery of rETR_max_ (Fig. 8b, Table 1). A significantly increasing α value after rehydration was only observed in discs with 48 % RWC (Table 1). However, the *α* value of control samples was never reached after rehydration (Table 1). Rehydration always led to a significant decrease of *I*_k_ and only in discs with 27 % RWC the initial value (control) was not reached again (Table 1).

**Table 1:** Characteristic photosynthetic parameters derived from rETR curves after desiccation for 30 min (RWC 73 %), 60 min (48 %) or 90 min (RWC 27 %) and rehydration in ASW for 2 h using the fitting model of Walsby (1997; *n* = 6, mean value ± SD)

Treatment	*α*	*I*_k_	rETR_max_
Control	0.39 ± 0.02^a^	53.18 ± 7.25^A^	18.17 ± 2.17^a^
30 min desiccation	0.24 ± 0.03^b^	122.48 ± 9.74^B^	25.32 ± 1.51^b^
+2 h rehydration in ASW	0.29 ± 0.07^+^	72.85 ± 13.74^*^	19.77 ± 3.91^*^
60 min desiccation	0.10 ± 0.02^c^	142.55 ± 22.27^B^	10.04 ± 2.06^c^
+2 h rehydration in ASW	0.28 ± 0.06^*+^	64.27 ± 24.27^*^	15.21 ± 2.70^*+^
90 min desiccation	0.05 ± 0.03^cd^	140.00 ± 21.01^B^	3.00 ± 1.94^d^
+2 h rehydration in ASW	0.06 ± 0.03^+^	106.77 ± 19.89^+^	5.99 ± 2.52^+^

Data were analysed by one-way ANOVA followed by Tukey’s post hoc test

Significant differences (*P* < 0.001) between control group and desiccated samples are indicated by small letters (*α*), capital letters (*I*_k_) and underlined letters (rETR_max_). Additionally, parameters of desiccated samples and recovered samples always were compared and significant differences are marked with an asterisk (*P* < 0.001). Furthermore, significant differences (*P* < 0.001) between control and rehydrated samples are marked with a plus

*α* Initial slope at light limiting photon fluence rates (electrons photon^−1^), *I*_k_ initial value of light-saturated photosynthesis (μmol photons m^−2^ s^−1^, *rETR*_*max*_ maximum electron transport rate (µmol electrons m^−2^ s^−1^)

## Discussion

In the present study of *U. compressa*, we found that upon desiccation the inner cell wall layers followed the water loss of the cells, which allowed cells to maintain turgor pressure, thus contributing to the ability of this algae to avoid mechanical damage. We believe that previously uncharacterized structural adaptation has a major influence on *U. compressa*, and likely in other species of *Ulva*, to accomplish DT, as the main organelles remain intact, and a recovery of photosynthesis is possible. In addition, investigations of the photosynthetic performance under desiccation allowed insight in the physiological limits of this organism, thus contributing to a better understanding of DT mechanisms in marine macroalgae.

### Phylogenetic position and morphology

Recent investigations have stressed the importance of connecting physiological and ecological studies of *Ulva* to strains that are accurately identified and precisely known (Kirkendale et al. 2013), because great differences in characteristics can be found among species and strains. In this study, we used both morphology and molecular phylogenetics to place the new strain as a member of *U. compressa* (formerly *Enteromorpha compressa*).

Although Blomster et al. (1998) consider branching as a primary morphological character of *U. compressa*, we never observed branching in our thalli. This is likely attributed to the constant salinity regime in the habitat and in culture (Burrows 1959). It was shown that unstable salinity regimes or salinity shocks are a key factor for the induction of branching in *Ulva* (Reed and Russel 1978). Branching often is accompanied by an arrangement of cells in short rows (Blomster et al. 1998). In our unbranched samples, the cells were irregularly arranged, often overlapping in surface view and sometimes organized into ‘cell islands’ where the cells appeared spherical, which was in good agreement to previous descriptions (Bliding 1963; Wolf et al. 2012). The tendency towards distortion of cell arrangement increased in older thalli was also reported for *Ulva flexuosa* subsp. *pilifera* (Messyasz et al. 2013). Moreover, an accompanying microbial flora is crucial for normal morphogenesis in *Ulva*, when experimentally changed unusual cell wall protrusions, loss of differentiation of rhizoid cells might be the consequence (Spoerner et al. 2012).

### Significance of cell wall components

The astonishing global success of *Ulva* in coastal habitats, likely promoted by high anthropogenic nutrient and heavy metal input (Veccia et al. 2012), has attracted scientists to investigate the underlying cellular mechanisms that contribute to such wide environmental tolerances. While several studies have focused on cell physiology (Longstaff et al. 2002; Carr and Björk 2003; Zou et al. 2007; Zhang et al. 2012; Mou et al. 2013; Gao et al. 2010, 2011, 2014, 2015), the role of cell wall components is less understood. Early TEM investigations provided a good description of the structural components (Bråten and Løvlie 1968; Micalef and Gayral 1972; Bråten 1975; McArthur and Moss 1977, 1978). These studies lacked further explanations of the cell wall’s contribution to maintain and re-establish physiological processes. We consider the cell wall properties as a major contributor to the DT mechanisms in *U. compressa*, and likely applicable to other Ulvophycean species.

In the present study, we focused on the structural nature of the cell wall, investigated by straightforward staining procedures to gain an understanding of the role of the different components during desiccation stress. We found that even in hydrated cells, the cell walls comprise different layers, where the outer layers (i.e. the border between individual cells) mostly contain filamentous structural components involving unbranched linear polysaccharides, which was shown by CFW staining. We are aware that also callose could be stained by this component as well (Krishnamurthy 1999); however, callose can be excluded in the case of *U. compressa* as aniline blue staining was negative (Fig. S2). The fibrillar material, mainly containing cellulose and xyloglucans (Lahaye and Robic 2007), acts as a framework for the incorporation of later synthesized amorphous cell wall material derived from the Golgi apparatus (McArthur and Moss 1977). This inner part of the cell wall is organized in several layers, which are formed by several distinct phases of generalized deposition of cell wall material (McArthur and Moss 1978). They can be clearly depicted by TEM images, which are in good accordance to previous studies in *Ulva* sp (Bråten and Løvlie 1968; Micalef and Gayral 1972; Bråten 1975; McArthur and Moss 1977). The innermost cell wall layers contain few load-bearing components and appeared more amorphous. This is clearly visible in our TEM images, and corroborated by the histochemical observations that both RR (which is wildly used to depict ‘pectic’ components) and TB stain these innermost cell wall layers. We also observed conspicuous electron translucent layers, which were mainly restricted to the periclinal cell walls and likely contain high amounts of glucuronan (Lahaye and Robic 2007).

Most interestingly, already after desiccation to a RWC of 73 % a dramatic change of the cell wall can be seen. TB-stained semithin cross sections show that parts of the outermost cell wall layers surrounding the cells fragment and remain attached as caps on the cell walls of individual cells. This glucuronan-rich cell wall area, which likely also contains ulvan (Lahaye and Robic 2007), might act as a matrix in which the individual cells surrounded by their cell walls are embedded. In contrast to the matrix, the dense fibrillar frame of the individual cells stays intact upon desiccation as shown by TEM, while less dense cell wall components enclosed by this frame appeared undulated. The pectin-rich innermost cell wall layers appear especially flexible. This allows a controlled shrinkage process of the cytoplasm during water loss, as parts of the cell wall follow the retraction of the protoplast. Furthermore, upon rehydration, the flexible cell wall layers contribute to an organized expansion process, which also is considered to be of prime importance in desiccation-tolerant plants (Moore et al. 2013). At all times of the desiccation and rehydration cycle, the plasma membrane is in close contact to the inner layers of the cell wall and no plasmolysis was found. This is particularly interesting, as plasmolysis was observed by light microscopy as well as a similar TEM fixation protocol in streptophyte green algae upon desiccation (Holzinger et al. 2011; Karsten and Holzinger 2012) and osmotic dehydration (Kaplan et al. 2012, 2013). Chemical fixation proved to be particularly suitable for analysing these structural and amorphous components of the cell walls (Domozych et al. 2007). The flexible glucuronan- and ulvan-rich matrix embedding the cells might also play a fundamental role in preventing disaggregation of the thallus during desiccation and rehydration cycles. Desiccation stress involves water removal from the apoplast, resulting in shrinkage of the whole thallus as shown by TB-stained semithin cross sections. This forces individual cells to shift against each other, while shearing forces leading to cell detachment might be absorbed by the matrix.

### Physiological performance

As the focus of the present study was on DT mechanism, we investigated physiological parameters in more detail. It has been found that *U. compressa* has a narrow light optimum as investigated by rETR, where above ~200 µmol photons m^−2^ s^−1^ photoinhibition was observed. This can likely be explained by the natural habitat, where excess light is only a problem for upper layers (Bischof et al. 2002b). Most interestingly, 30 min of desiccation, corresponding to a RWC of about 73 %, has a stimulating effect on rETR kinetics. In fact, rETR_max_ was significantly enhanced from 18.17 ± 2.17 to 25.32 ± 1.51 µmol electrons m^−2^ s^−1^ (Table 1).

This observation might be explained by a general stimulation of the antioxidative systems, which are beneficial for light stress, desiccation stress and even copper stress (Kranner and Birtic 2005; Contreras-Porcia et al. 2011; Zhang et al. 2012; Xie et al. 2013). Recently, Zhang et al. (2012) used a *de novo* sequencing transcriptomic approach in the closely related *Ulva linza* to show a strong ability for enzymatic ROS detoxification by, e.g. superoxide dismutases (SODs), ascorbate peroxidase (APX) and catalase (CAT). Hypersalinity and hydrogen peroxide lead to an up regulation of gene expression in antioxidative enzymes in *Ulva fasciata* (Sung et al. 2009). In nature, *Ulva* sp. growing in intertidal areas are permanently exposed to these fluctuations (Gao et al. 2011). In this context, it might be additionally beneficial that electron flow in PS I is stimulated by mild desiccation conditions in *Ulva* sp. from the Yellow Sea (Gao et al. 2011). Moreover, high light as well as desiccation increased light harvesting proteins such as LHCSR and PsbS (Mou et al. 2013; Gao et al. 2015), which play an essential role in non-photochemical quenching (NPQ) and the protection of PSII (Roach and Krieger-Liszkay 2014). This is beneficial particularly for the intertidal alga *Ulva*, as desiccation stress always goes along with high irradiance, when the thalli are not protected by a water layer (Bischof et al. 2002b). However, under more severe desiccation conditions (i.e. 27 % RWC), we showed a drastic decrease of rETR_max_. Measurable rETR were still observed, suggesting that *U. compressa* can tolerate this level of desiccation. The PS II dependent rETR might be related to the recently demonstrated selective influence of DT on the different photosystems in *Ulva* (Gao et al. 2011); in particular, the PS I is less perturbed by desiccation and functions even at RWC of 22 % (Gao et al. 2011). These findings may shed light on our observations that electron flow is still measurable (positive rETR values), but with a drastic decrease, at 27 % RWC in *U. compressa*.

Salt stress was avoided by removing excess of ASW prior to the desiccation treatments. It was evident, that even the 90-min desiccated samples, despite showing a reduction in chlorophyll *a* and *b* content, still appear visibly green. This is corroborated by findings of Xia et al. (2004) who showed that the contents of chlorophyll a, b and carotenoids during short-time exposure to salt stress were unaffected in *Ulva lactuca*. In contrast, the crucial factor for pigment reduction is light intensity (Hernández et al. 1997), which was excluded by our experimentation performed under constant low light regime.

We aimed to determine if the photosynthetic parameters changed across the thallus segment simultaneously, or if there is a gradual decrease of e.g. *F*_v_/*F*_m_ from the outside to the inside. Therefore, we applied for the first time a combination of an Imaging PAM with a microscope. This allowed us to investigate the *F*_v_/*F*_m_ value at a cellular level, demonstrating that the decrease of the maximal photosystem II efficiency is more or less uniform over a thallus segment with a diameter of 6 mm. We are aware that the measured *F*_v_/*F*_m_ values from the Imaging-PAM system are slightly lower than those generated by the PAM-2500 system, but this is likely due to the combination of a microscope, the aperture of the objective lens and the different excitation wavelengths of these instruments (PAM-2500: 630 nm; Imaging PAM: 620 nm). Moreover, this resolution of the *F*_v_/*F*_m_ value demonstrates that within the thalli the desiccation-induced reduction of the *F*_v_/*F*_m_ values was homogenous and we conclude from these observations that all of the examined cells were in a similar physiological state. Gao et al. (2011) showed a similar arrangement of the *F*_v_/*F*_m_ value within the thalli of *Ulva* sp. using the MAXI version of an Imaging PAM. However, they did so at a markedly lower resolution and they were not able to monitor the condition of individual cells.

Full recovery of *F*_v_/*F*_m_ after rehydration in ASW for 2 h was only observed in samples desiccated for 30 min. However, the *F*_v_/*F*_m_ value was drastically reduced after 90 min of desiccation (~0.1), and did not recover which points towards an irreversible change of PS II. In contrast, the rETR_max_ values still tend to recover after 90 min desiccation. This might be explained by the different experimental setup for *F*_v_/*F*_m_ and rETR_max_ measurements (Fig. S1). Whereas the *F*_v_/*F*_m_ was determined by repeated measurements of the same samples, the rETR curves were recorded independently in control, desiccated and rehydrated samples. Repeated saturation pulse analyses (for *F*_v_/*F*_m_ determination) can be regarded as high light stress leading to full reduction of the plastoquinone pool (Baker 2008). This is particularly harmful to cells already suffering from water limitation. Findings in the present study were obtained from laboratory experiments, which explain the lower capacity to recover PSII efficiency during rehydration compared to field-collected *Ulva* sp. (Gao et al. 2011). Naturally grown populations of *Ulva* likely undergo hardening processes that increase their ability to recover after desiccation stress, which was also discussed recently in aero-terrestrial streptophyte green algae (Pichrtová et al. 2014a, b; Herburger et al. 2015). Formation of cell walls in *Ulva* seems to be an ongoing process, which explains that the inner cell walls of some cells of *Ulva intestinalis* comprised of 16 layers surrounded by additional 5 outer cell wall layers (McArthur and Moss 1977).

We conclude that structural changes in the cell walls of *U. compressa* during desiccation–rehydration cycles play a key role in surviving high and low tides. While short-term desiccation can lead to a stimulation of photosynthesis, longer water stress involves a reduction of photosynthesis indicated by decreasing *F*_v_/*F*_m_ and rETR_max_ values. Future investigations of antibody labelling of cell walls would deepen our understanding of the different cell wall layers (e.g. sulphated polysaccharides, differentiation within pectin-rich components such as homogalacturonan, rhamnoxylogalacto-glucuronan). These investigations were beyond the scope of the present study, but will certainly elucidate the mechanical properties of the different cell wall layers. This will help us to get a deeper insight  into the role of the cell wall properties in DT of intertidal algae.

#### *Author contribution*

A.H. planned experiments, performed experiments, wrote manuscript, K.H. performed experiments, wrote manuscript, F.K. performed experiments, L.A.L. performed experiments, wrote manuscript.

## Electronic supplementary material


**Fig. S1** Experimental setup for controlled desiccation and rehydration of *Ulva compressa* thallus discs for microscopic and physiological investigations. After setting the RH to ~62 % inside the chamber the discs were desiccated for 30, 60 or 90 min. The *F*_v_/*F*_m_ values were measured in three time series: control, desiccated (30, 60 or 90 min) rehydrated discs. rETR values were determined independently for control, desiccated (30, 60 and 90 min) and rehydrated discs (*dashed line*). The relative water content for each desiccation level is shown (TIFF 90 kb)

**Fig. S2** Aniline blue staining of *Ulva compressa*. **a** Surface view of a thallus segment (DIC). **b** Fluorescence image of the same area. Chloroplast autofluorescence is shown (*red*). The cell walls lack callose as they are not stained by aniline blue. *Scale bars* 10 µm (TIFF 2142 kb)

**Fig. S3** Chlorophyll *a*, *b* and carotenoids of control discs and discs desiccated for 30, 60 or 90 min (*n* = 4). Significances between the groups are indicated by *small letters* (chl. *a*), *capital letters* (chl. *b*) and *underlined letters* (carotenoids). They were determined by one-way ANOVA (*P* < 0.001) followed by Tukey’s post hoc test (TIFF 72 kb)

## Abbreviations

AB: Aniline blue
AIC: Akaike information criterion
*α*: Positive slope at limiting photon fluence rates
APX: Ascorbate peroxidase
ASW: Artificial sea water
CAT: Catalase
CFW: Calcofluor white
*F*_m_: Maximal fluorescence yield from dark-adapted material
*F*_o_: Basic fluorescence yield from dark-adapted material
*F*_v_/*F*_m_: Maximum photochemical quantum yield of PSII
*I*_k_: Initial value of light-saturated photosynthesis
LhcSR: Light-harvesting complex stress-related
ML: Maximum likelihood
NA: Numerical aperture
PAM: Pulse amplitude modulation
PAUP: Phylogenetic analysis using parsimony
PSII: Photosystem II
*rbc*L: Large subunit of rubisco
rETR_max_: Maximum relative electron transport rate
RH: Relative humidity
ROS: Reactive oxygen species
RR: Ruthenium red
RWC: Relative water content
SOD: Superoxide dismutase
TB: Toluidine blue
TEM: Transmission electron microscope
*W*_*0*_: Wet weight
*W*_*d*_: Dry weight
*W*_*t*_: Weight after *t* desiccation
