# Supplementary figures and images for: Desiccation tolerance in the chlorophyte green alga Ulva compressa: does cell wall architecture contribute to ecological success?
Source: Planta. 2015 Apr 21;242(2):477–92. doi: 10.1007/s00425-015-2292-6 (PMC4498240; doi:10.1007/s00425-015-2292-6)

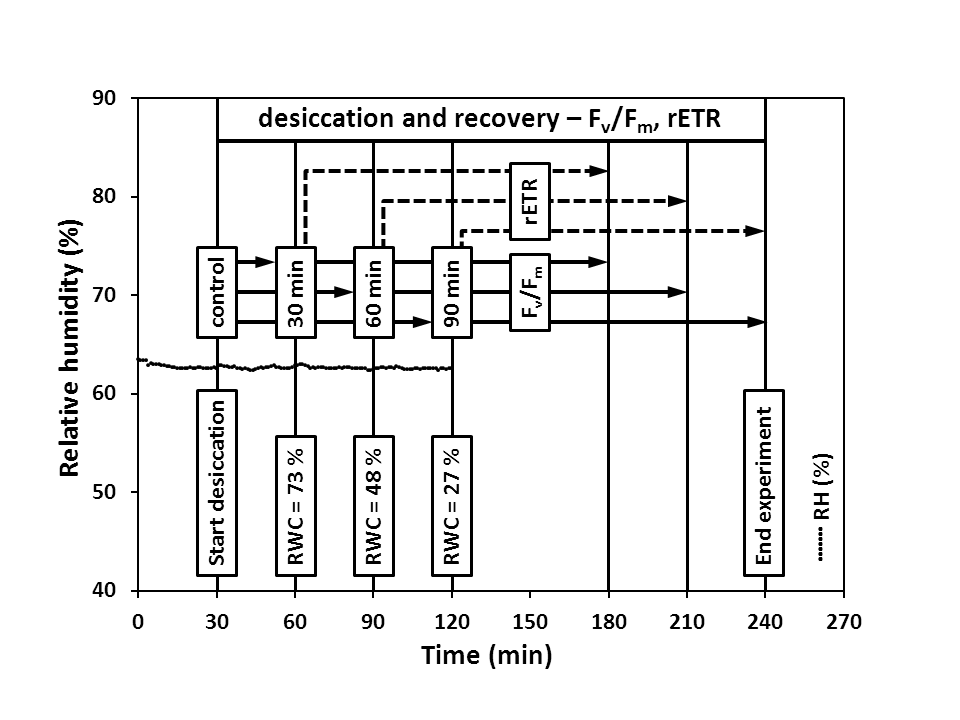

Supplement: Supplementary file 1 — Fig. S1 Experimental setup for controlled desiccation and rehydration of Ulva compressa thallus discs for microscopic and physiological investigations. After setting the RH to ~62 % inside the chamber the discs were desiccated for 30, 60 or 90 min. The F v/F m values were measured in three time series: control, desiccated (30, 60 or 90 min) rehydrated discs. rETR values were determined independently for control, desiccated (30, 60 and 90 min) and rehydrated discs (dashed line). The relative water content for each desiccation level is shown (TIFF 90 kb) [file 425_2015_2292_MOESM1_ESM.tif]

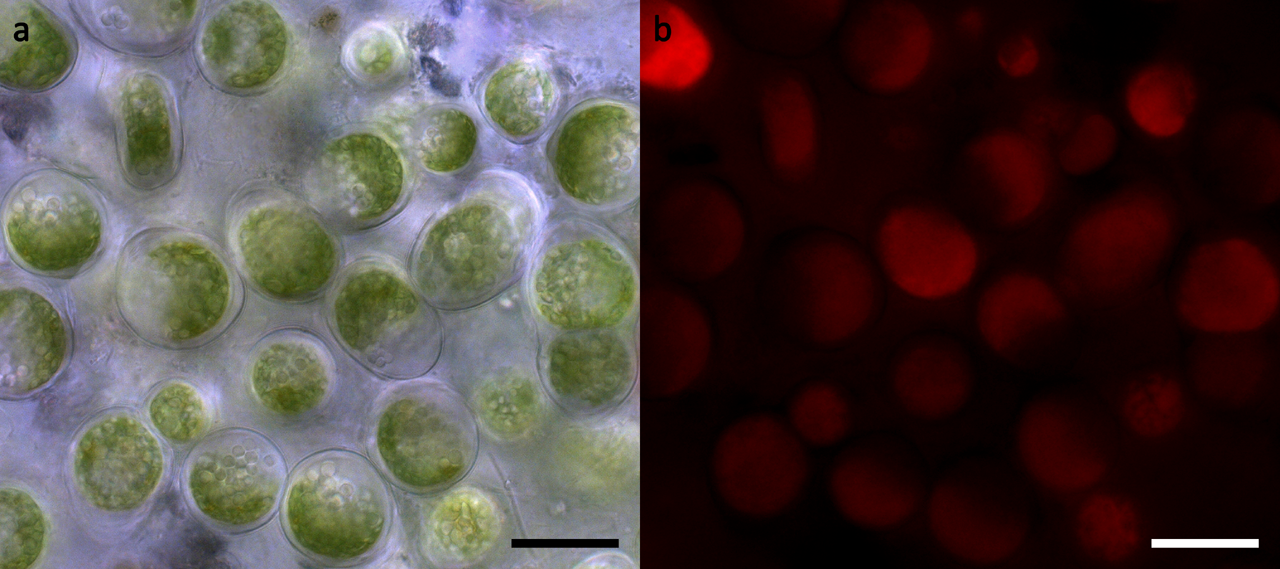

Supplement: Supplementary file 2 — Fig. S2 Aniline blue staining of Ulva compressa. a Surface view of a thallus segment (DIC). b Fluorescence image of the same area. Chloroplast autofluorescence is shown (red). The cell walls lack callose as they are not stained by aniline blue. Scale bars 10 µm (TIFF 2142 kb) [file 425_2015_2292_MOESM2_ESM.tif]

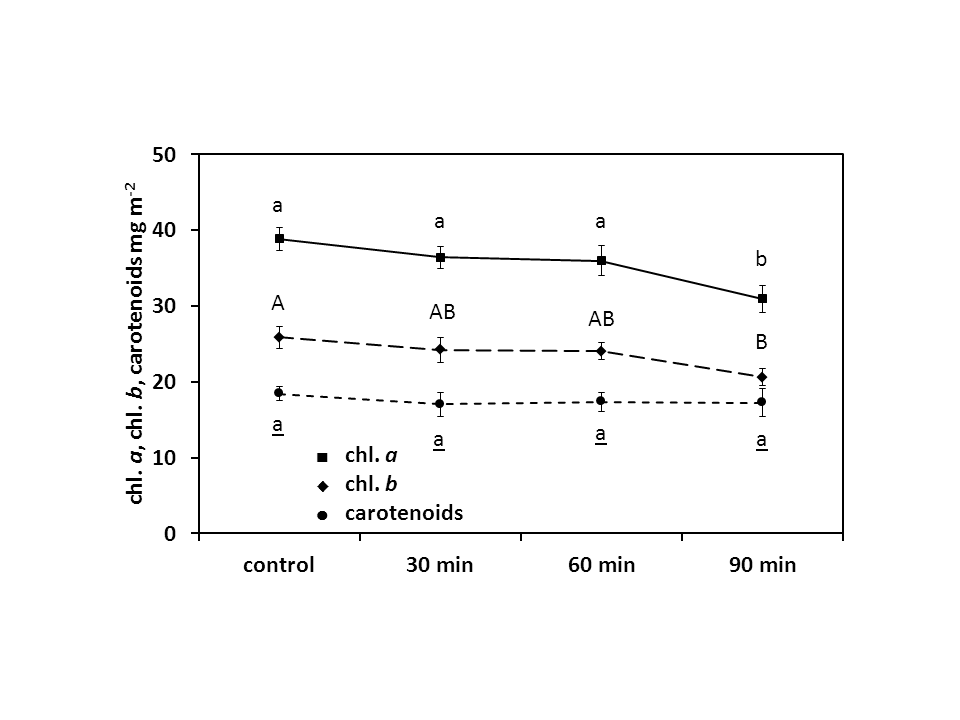

Supplement: Supplementary file 3 — Fig. S3 Chlorophyll a, b and carotenoids of control discs and discs desiccated for 30, 60 or 90 min (n = 4). Significances between the groups are indicated by small letters (chl. a), capital letters (chl. b) and underlined letters (carotenoids). They were determined by one-way ANOVA (P < 0.001) followed by Tukey’s post hoc test (TIFF 72 kb) [file 425_2015_2292_MOESM3_ESM.tif]
